# Impact of individualized colored spectacle filters on photophobia and visual comfort in central visual field defect patients: a one-year study

**DOI:** 10.1038/s41598-026-45302-w

**Published:** 2026-03-26

**Authors:** Margarita Krasniakova, Tony Pansell, Jörgen Gustafsson

**Affiliations:** 1 Division of Eye and Vision, Department of Clinical Neuroscience, Karolinska Institutet, 17177 Stockholm, Sweden; 2 St. Erik Eye Hospital, 17164 Stockholm, Sweden; 3 Department of Optometry, Radiography and Lighting Design, University of South-Eastern Norway, Campus Kongsberg, 3603 Kongsberg, Norway

**Keywords:** Diseases, Health care, Medical research, Neuroscience

## Abstract

Visual comfort is a critical yet often overlooked aspect in managing patients with central visual field defects and photophobia. This study investigates the long-term effects of individualized colored spectacle filters on visual comfort and function in patients with Age-related Macular Degeneration and Leber Hereditary Optic Neuropathy. Patients were fitted with individualized precision tint spectacles tailored to improve vision comfort for one year. The study involved a comprehensive evaluation of visual acuity, contrast sensitivity, and photophobia symptoms alongside qualitative feedback from patient interviews. Results indicate significant improvements in subjective visual comfort and function, with a notable reduction in photophobia symptoms among patients with Leber Hereditary Optic Neuropathy. Colored filters, particularly those in the blue-green spectrum, were preferred by most patients and had a positive impact on comfort. Statistical analysis revealed trends in color preferences and subjective enhancements in visual function, underscoring the potential benefits of comfort-tint filters and the importance of personalized treatment approaches. The findings suggest that individualized colored spectacle filters can be valuable in enhancing visual comfort and function for patients with central visual field defects. This study shows the importance of integrating visual comfort considerations into clinical practice for a comprehensive approach to patient care.

## Introduction

Visual comfort is often overlooked in eye care. Most effort is given to visual functions that can be objectively valued, such as visual acuity, contrast sensitivity, and near-visual functions, such as reading. The decision to provide a treatment or sell optical correction depends on whether vision will improve objectively. One reason might be discomfort from uncorrected refractive errors^1^, accommodative- and convergence dysfunction^2,3^ that is most often reduced or eliminated with spectacles or vision-optimizing therapy in the healthy population. Additionally, most people have a preconceived notion of spectacles, which are used to enhance visual function. Still, discomfort is a common complaint following eye diseases and frequently remains troublesome for the patient despite optical correction. According to our clinical experience, a common symptom of eye diseases with central visual field reduction is ocular discomfort, mental fatigue, and tiredness when performing visually demanding tasks, which reduces working capacity. The mechanisms behind these complaints remain unclear. Symptom severity might depend on the degree of visual reduction in acuity, visual fields, or contrast sensitivity. Poorer sensory input has been shown to increase the cognitive effort needed to compensate for the reduced signal-to-noise ratio in hearing^4^, according to the principles of compensatory cortical recruitment. A similar principle is also plausible for a reduced visual signal. Impaired depth perception or the reliance on only peripheral vision can make navigating spaces and locating objects in a cluttered environment more challenging. Also, visual attention must be shifted from the habitual central part of the field into a preferred retinal location, allowing cognitive resources to elaborate on the fixated object of interest.

Biological changes in eye diseases can also cause tired eyes or ocular discomfort. Photophobia is a common symptom in ophthalmic disorders, leading to mild to severe discomfort, even pain. Retinal melanopsin receptors (ipRGC) have been found to play a key role in photophobia. They are most sensitive to blue light, particularly around 480 nm, which corresponds to the absorption peak of the photopigment melanopsin^5^. ipRGC can activate nociceptors, potentially contributing to light-induced pain without involving the retina and optic nerve^6^. A previous study demonstrated that filters blocking wavelengths around 480 nm reduced mean blink rate more than standard sunglasses in blepharospasm patients^7^, a condition with photophobia. Additionally, short wavelengths have been shown to reduce activation in several brain areas in patients with chronic ocular pain^8^. It is plausible to hypothesize that melanopsin photoreceptors could influence photophobia in patients with central visual field loss^9^. There are several questionnaires developed to evaluate the impact of photophobia, like the 8-item Korean Photophobia Questionnaire (KUMC-8)^10^, the 17-item Utah Photophobia Questionnaire (UPSIS-17)^11^, and the Visual Light Sensitivity Questionnaire-8 (VLSQ-8)^12^. We have chosen to use the VLSQ-8 in this study.

Optical color filters have traditionally been prescribed to optimize contrast sensitivity in low vision^13,14^ and to reduce photophobia and glare. Selective filters blocking short-wave blue light, known as edge filters, have been shown to improve acuity and contrast sensitivity in diabetic retinopathy^15^ and to enhance the quality of life in individuals with Age-related Macular Degeneration (AMD)^16^. A similar study on AMD patients found no enhancement in acuity or contrast^17^. Newer selective filters, often referred to as comfort filters, suppress more light in the middle of the color spectrum or filter out long-wave light. Testing is performed by holding hand-held flippers or filters mounted in the trial frame, using ready-made filters supplied by the manufacturer. With the introduction of the Intuitive Colorimeter (Cerium Visual Technology, United Kingdom), a more systematic approach to investigating color filter preferences is available. The Intuitive Colorimeter is a device that determines the individual’s optimal color preference. It was developed by Professor Arnold J. Wilkins and described first in 1992 in a technical note^18^. It allows for the precise adjustment of color, brightness, and saturation. The measurement result provides for ordering lenses with precise individualized tints, so-called precision tints.

Central visual field reduction, characterized by the loss or decrease of visual function in the central part of the visual field, profoundly impacts activities requiring detailed vision, such as reading, writing, and recognizing faces, and influences the quality of life^19–21^. AMD is the leading cause of low vision in the aging population in the Western world^22^. Visual acuity in patients with AMD can vary widely, depending on the stage and type of the disease. Patients may experience subtle visual disturbances in the early stages, while in the advanced stages, particularly in the neovascular form, it can lead to severe central vision loss^23^. The presence of AMD in one eye significantly increases the risk of developing the condition in the other^24^. In contrast, Leber Hereditary Optic Neuropathy (LHON) is a rare mitochondrial disorder typically affecting young males. This visual loss typically begins rapidly and painlessly; most patients deteriorate to visual acuities worse than 20/200, and the second eye is usually affected within weeks to months, with 97% of patients experiencing this within one year^25^.

This study aimed to elucidate the concept of visual comfort in patients with central visual field reduction by testing the effect of colored filters and fitting participants with precision tint spectacles that included refraction correction for use over one year. Two groups are involved: one group with LHON and one group with AMD.

Research questions are: Do patients prefer precision tint filters to previously worn filters from the low vision clinic, and if so, why? Is there a preference for a particular color for LHON and AMD? Are vision-optimizing filters and comfort-optimizing filters the same? Do comfort filters influence vision function, vision comfort, or both?

## Results

Ten patients with AMD and eleven with LHON were included in the study and completed the initial visit. One patient with AMD denied any positive effect when testing the comfort-tint in the examination room due to postural discomfort and asked to be excluded from the study. The analysis of color preferences for comfort-tint by eye disease will be calculated from nine patients with AMD (5M/4F, 78.3y (s.d. 7.9)) and eleven with LHON (8M/3F, 43.8y (s.d. 20.8)). For details of the testing protocol and colorimetry procedure, see the methods section.

At the follow-up visit after one year, four patients with AMD and one with LHON did not return. Two AMD patients died during the study period from causes not related to their eye disease. One AMD patient came for follow-up but experienced reduced visual acuity during the study period and was found to have corneal dystrophy. One AMD and one LHON patient did not respond to repetitive calls and were excluded from the study. Repeatability of comfort-tint measures and the effect of vision-tint will be calculated on five patients with AMD (4M/1F, 76.9y (s.d. 7.8)) and ten patients with LHON (7M/3F, 47.2y (s.d. 20.3)).

### Color hue preferences

Hue values and saturation levels were determined using the Intuitive Colorimeter as described in the methods section. The ’Rayleigh test of uniformity’ checks whether circular data is uniformly distributed around the circle (i.e., no preferred direction). The data display non-uniformity in all test conditions (Comfort 1 and Vision,* p*> 0.01; Comfort 2,* p* = 0.05), indicating that participants selected hues in specific directions rather than randomly.

**Fig. 1:**
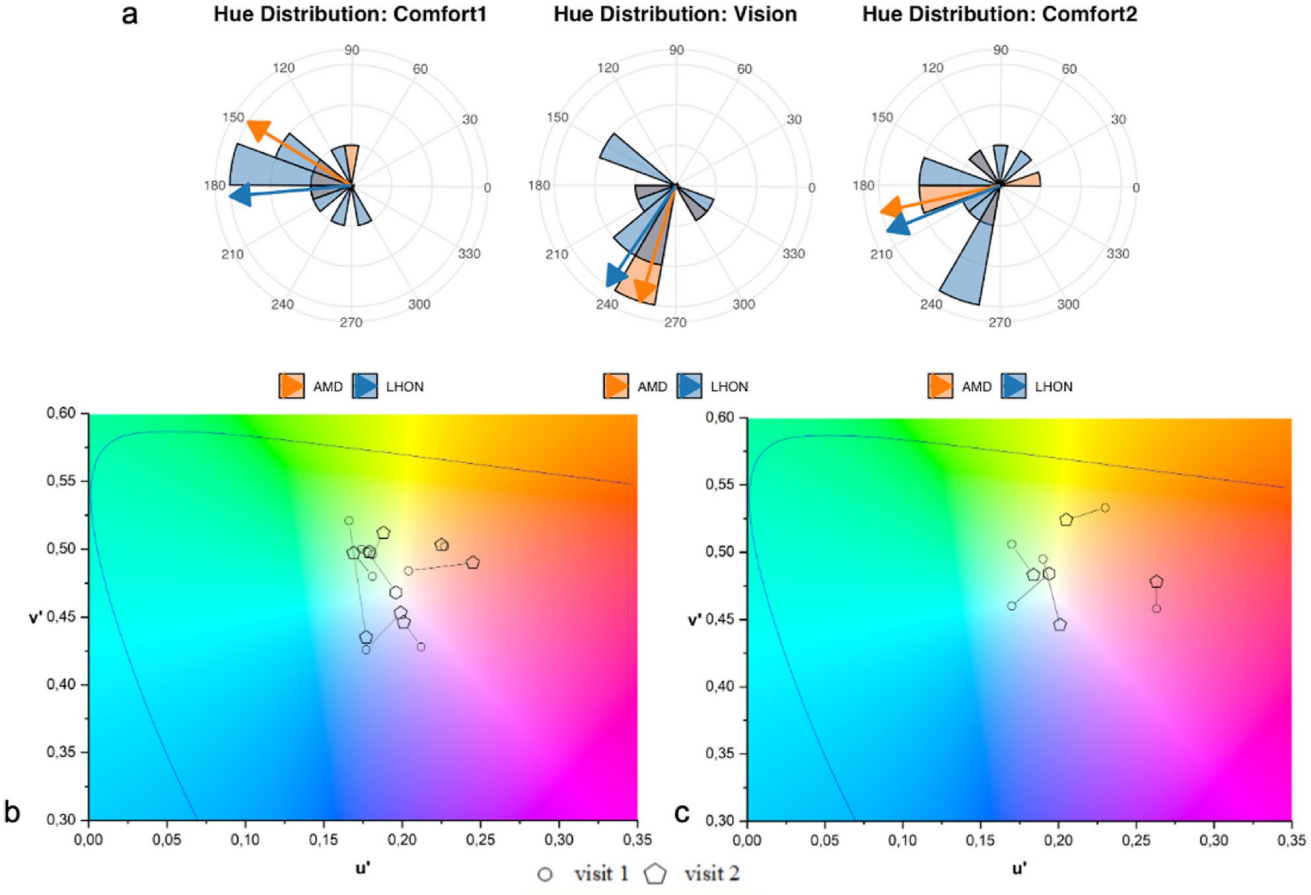
Distribution of preferred hues for each condition (**a**). Arrows display angular means per group (AMD = orange; LHON = blue). Individual-based repetability in CIE UCS (LHON = **b** and AMD = **c**).

Both groups tended to choose green-turquoise hues (150–200) for enhanced comfort, while enhanced vision revealed bluish hues (230–260). For details, see Table 1 and Fig. 1.

**Table 1:** Hue, saturation, and $\Delta u^{\prime }v^{\prime }$ for LHON and AMD, n.s. = not statistically significant.

Group	Measure	Visit 1	Visit 2	Vision tint Visit 2	Comfort tints $\boldsymbol{\Delta u^{\prime }v^{\prime }}$	Comfort–vision tints $\boldsymbol{\Delta u^{\prime }v^{\prime }}$
LHON (n=10)	Hue (deg)	185 (52), n.s.	202 (71), n.s.	236 (58), n.s.	0.030 (0.024)	0.049 (0.032)
	Saturation (%)	30 (7)	28 (4), n.s.	28 (4), n.s.		
AMD (n=5)	Hue (deg)	149 (70)	193 (75), n.s.	253 (33), n.s.	0.033 (0.01)	0.036 (0.02)
	Saturation (%)	30 (7)	26 (6), n.s.	28 (5), n.s.		

Values represent the circular mean hue angle (in degrees) and circular standard deviation (in parentheses) for Comfort 1, Comfort 2 and Vision tints. Saturation values are expressed as mean percentage (SD). The $\Delta u^{\prime }v^{\prime }$ values represent chromaticity differences in the CIE 1976 UCS coordinate space, calculated as Euclidean distances between the chromaticities of the tints. Specifically, “Comfort tints” correspond to the chromaticity shift from Comfort 1 to Comfort 2, while “Comfort–Vision tints” represent the chromaticity shift from Comfort 2 to the Vision tint

The repeatability of the comfort-tint in the LHON group showed no statistically significant difference $[W = 2.68,\ df = 2,\ p = 0.262]$. The average hue at the first visit was 185° (SD = 52), with an average saturation level of 28% (SD = 4). After approximately one year (mean = 371 days, SD = 26), at the second visit (i.e., comfort-tint 2), the average hue was 202° (SD = 71), with the same saturation of 28% (SD = 4), resulting in an average shift of 17°.

A larger but non-significant shift was observed in the AMD group, with an average hue of 149° (SD = 70) at the first visit and 193° (SD = 75) at the follow-up visit (mean = 352 days, SD = 37), corresponding to an average shift of 44° $[W = 0.28,\ df = 2,\ p = 0.872]$. See Table 1 for a graphical representation of angular hue values.

All participants underwent colorimetry testing to optimize their vision. The LHON group’s average hue for the vision-tint was 236° (SD = 58), and saturation was 28% (SD = 4). In comparison with the comfort-tint obtained on the same day (Comfort 2), no significant difference was found $[W = 0.12,\ df = 2,\ p = 0.944]$.

The AMD group’s average hue for the vision-tint was 253° (SD = 33), and saturation was 28% (SD = 5). In comparison with Comfort 2, no significant difference was found $[W = 1.72,\ df = 2,\ p = 0.424]$.

### The effect of tints and glare on visual acuity and contrast acuity

Visual acuity and contrast sensitivity were assessed using ETDRS charts under standardized conditions, as detailed in the methods section. Visual acuity (logMAR) was assessed at 100% and 10% contrast levels, with and without comfort-tint, and in the presence or absence of glare (only at the 100% level). All variables met assumptions for normality (Shapiro–Wilk $p > 0.135$ for all conditions), confirmed by inspection of Q–Q plots.

There was no significant main effect of condition (tint or glare) on visual acuity $\textrm{F}(3,43) = 0.912,\ p = 0.444$. At 10% contrast, a similar pattern emerged: the main effect of condition (tint/glare) was not significant $\textrm{F}(3,24) = 1.27,\ p = 0.424$. A significant main effect of disease group was found for both contrast levels – 100% $\textrm{F}(1,44) = 7.331,\ p = 0.0095$ and 10% $\textrm{F}(1,24) = 11.515,\ p = 0.002$ – indicating that patients with LHON, on average, demonstrated worse visual acuity. For details, see Table 2.

**Table 2:** ETDRS visual acuity (logMAR) for LHON and AMD.

Contrast level	1st visit – comfort-tint		2nd visit – vision-tint	
	No filter	Filter	No filter	Filter
LHON (n=10)				
100%, no glare	1.04 (0.63)	1.08 (0.63)	1.07 (0.65)	1.05 (0.65)
100%, glare	1.08 (0.63)	1.06 (0.65)	1.09 (0.67)	1.09 (0.67)
10%, no glare	1.75 (0.51)	1.68 (0.44)	1.66 (0.51)	1.67 (0.49)
AMD (n=5)				
100%, no glare	0.66 (0.15)	0.69 (0.18)	0.73 (0.15)	0.73 (0.14)
100%, glare	0.67 (0.15)	0.66 (0.18)	0.70 (0.14)	0.78 (0.06)
10%, no glare	1.16 (0.20)	1.18 (0.20)	1.25 (0.26)	1.24 (0.26)

Values represent mean logMAR visual acuity (standard deviation in parentheses) for participants with LHON (n=10) and AMD (n=5) under different contrast and glare conditions, with and without optical filters. Measurements were taken during the first visit (Comfort-tint) and second visit (Vision-tint). Lower logMAR values indicate better visual acuity.

A repeated measures ANOVA was conducted to evaluate the effect of vision tint. There was no significant main effect of condition (tint or glare) on visual acuity, $F(3, 48) = 0.000, p = 1.000$. The main effect of the condition (vision-tint use) was also not significant for the 10% contrast level, $F(1, 24) = 0.000, p = 1.000$. A significant main effect of the disease group was found for both contrast levels: 100% contrast, $F(1, 48) = 7.331, p = 0.009$, and 10% contrast, $F(1, 24) = 11.515, p = 0.002$. Patients with LHON demonstrated worse visual acuities on average compared to those with AMD.

### Patient-reported outcomes from VLSQ-8 and interviews

All participants completed the VLSQ-8 questionnaire regarding photophobia at the initial visit, prior to using the comfort-tints, and at the follow-up visit one year later. Patients with LHON generally had more photophobia symptoms compared to the AMD group. The AMD group displayed a non-significant reduction of symptoms with 15.0 (s.d. 5.9) before and 12.8 (s.d. 5.1) when using the comfort-tints ($z = 0.809; p = 0.498$). The LHON group displayed a significant reduction of symptoms from a median score of 23.2 (s.d. 7.1) before use and with comfort-tints of 19.0 (s.d. 6.2); $z = 2.521; p = 0.014$.

The participants reported their use and experience with spectacles with comfort-tints. On average, AMD participants used the comfort-tints more frequently (mean = 5.4 days/week, SD = 2.3) and for longer durations (mean = 4.6 hours/day, SD = 2.2) compared to LHON participants (mean = 2.6 days/week, SD = 2.2; mean = 3.7 hours/day, SD = 4.2). A Mann-Whitney U test revealed that AMD participants were significantly more likely to report improved vision with comfort-tint ($U = 40, p = 0.037$). LHON participants reported improved comfort more frequently ($U = 15, p = 0.049$). AMD participants, however, were more likely to choose tints for improved comfort rather than improved vision ($U = 42.5, p = 0.016$). Participants were positive about paying for the spectacles and continuing to use them after the study period. No significant differences were found between groups.

Thematic analysis of open-ended responses revealed distinct usage patterns and motivations between groups. The LHON participants primarily used the comfort-tints outdoors or continuously, citing reasons such as “improved comfort,” better appearance, and social concealment.” They described discomfort or glare when removing the comfort-tints and emphasized that the filters provided “specific” or “more exact” comfort compared to previous filters from the low-vision center. AMD participants used comfort-tints during specific tasks such as watching TV or working outdoors, emphasizing improved vision and reduced squinting. They noted temporary blur or color shifts when removing the filters, but described comfort-tints as more comfortable and less dark than traditional filter glasses from the low-vision clinic.

## Discussion

This study aimed to elaborate on visual comfort in patients with central visual field reduction. None of the participants had prior experience of testing comfort as part of a visual examination. On the other hand, everyone had some degree of photophobia due to their eye disease, was aware of discomfort, and understood the concept of optimizing this separately from vision. With many years of experience in testing traditional ready-made filters, the Colorimeter routine helps in evaluating the effect of filters and selecting the individual filter color in a structured manner. Being able to test the filter combination directly was greatly appreciated by patients and examiners. All participants except one with AMD found comfort-tints to improve comfort at the first visit and were willing to use comfort-filter spectacles during the study period. The reason why the excluded patient experienced dizziness is not apparent. The comfort effect is thus not purely a reduction of luminance, like a pair of neutral grey sunglasses that reduces the entire color spectrum. A typical comment in the follow-up interview was that the brighter comfort-tint lenses were more effective in improving comfort compared to the previously worn sunglasses or edge filter lenses from the low-vision clinic. This finding suggests that the improved comfort is not a result of reduced luminance, but a chromatic effect where the triggering colors of the light spectrum are reduced.

Based on the clinical experience of the low vision group, we know that filters rarely improve visual acuity but sometimes enhance contrast acuity, which agrees with previous studies in AMD^17^. Here, the measure was taken to be correlated to the subjective estimation of comfort. An improved acuity with filters could alleviate some of the cognitive load that patients with reduced vision experience, due to a similar mechanism as previously described by Peelle et al.^4,26^ A reduced function of the central visual field requires visual searching and attention over a longer period to complete a visual task. If filters improved visual acuity and contrast, a reduced discomfort could hypothetically be expected. Here, we could not demonstrate any objectively improved visual function; instead, the opposite was observed in several cases, even though they reported improved vision with comfort tints, especially in the group with AMD. One obvious reflection on this finding is how we clinically assess vision in patients with low vision. We traditionally rely on ‘objective measures’ of visual acuity and draw conclusions and provide recommendations based on these. Still, we miss something vital since we cannot explain or verify the subjective perception of improved vision. Could comfort-optimizing filters optimize higher vision functions, reducing the effort needed to perceive or to complete a visual task?

The chosen colors to optimize comfort were not randomly distributed on the color wheel but tended to cluster toward the green-turquoise spectrum (towards 180 degrees) for both patient groups. This is in the opposite direction to what is typically prescribed at the low vision clinic when optimizing contrast with yellow filters (towards 60 degrees). An even more unusual finding was observed during the second visit when optimizing vision in the Intuitive Colorimeter. Both patient groups chose blueish filters, that is, in the opposite direction to yellow (towards 240 degrees). Only one patient with LHON and one with AMD chose a yellow tint for improved comfort; none chose a yellow filter for improved vision. This challenges current filter testing practices. The yellow filter reduces short-wavelength light, thereby enhancing the contrast and optical quality of the retinal image. Nearly all patients had or had been offered yellow filters at the low vision clinic. Only a few used them. None had been tested with blueish or greenish filters. Blue-colored filters (not blue-blocking filters) are viewed with scepticism within eye healthcare since ultraviolet radiation (outside the visible spectrum) is well-documented to increase the likelihood of several eye diseases like cataract^27^, keratoconus^28^ or AMD^29^. Additionally, blue light (in the visible spectrum) from screens is known to disrupt the circadian rhythm^30^, affecting sleep patterns and quality. For this reason, blue-filters are today integrated into mobile devices and screens and offered as blue-light protection in computer glasses. Notably, that polycarbonate lenses in spectacles provide high protection from UVA and UVB radiation, even without any coating. This is in contrast to the standard plastic lens (CR-39) that needs surface coating to give full UVA protection^31^. Blue visible light is, however, still transmitted through both lens types and can disrupt the circadian rhythm.

Where in the ocular-visual system the increased comfort originates from is not clear. Our study groups have diseases in the retina and optic nerve, and both diseases suffer from photophobia. Is it possible that photophobia and discomfort are inherently connected and that our search to optimize comfort is merely an effect of reducing the light-spectrum triggering photophobia? Tinted lenses are prescribed for several medical conditions, including migraine^32–34^, mTBI^35,36^, blepharospasm^7,37^, visual stress^38^, and ophthalmological conditions affecting the eye, among others. All conditions have photophobia as part of the symptom constellation. Recent studies highlight the significance of ipRGC (intrinsically photosensitive retinal ganglion cells) in explaining the connection between photosensitive cells in the eye and the neural pain system^39^. The ipRGCs have a maximum sensitivity in the short-wave spectrum^5^, and the pink-reddish FL-41 filters have their maximum filter effect close to this and are known to alleviate photophobia in several of the conditions mentioned. However, this does not align well with our findings on green-turquoise filters to optimize comfort; our groups preferred filters in the green-blue spectrum that do not primarily reduce in the short-wave spectrum and therefore doesn’t target the ipRGC primarily. This necessitates another, or complementary, explanation to understand the concept of vision comfort in low-vision patients.

An alternative explanation for the observed preference for green-turquoise hues (180°), despite their proximity to the melanopsin sensitivity peak (480 nm; approximately 250–260° on the color wheel), may lie in the underlying mitochondrial pathology present in both LHON and AMD^40^. Prolonged exposure to blue light in rats led to mitochondrial damage in the inner retina, particularly in the axons and dendrites of retinal ganglion cells^41^. If similar damage occurs in human ipRGCs or other light-sensitive cells due to disease-related mitochondrial dysfunction, it is plausible that these cells may become less responsive to their typical activating wavelengths. In this context, patients may be compensating for a loss of sensitivity in the blue spectrum by selecting filters that enhance stimulation in that range, effectively attempting to restore a perceptual or physiological input that has been diminished due to cellular degeneration. This compensatory mechanism could coexist with, or even override, the typical aversive response to short-wavelength light, resulting in a paradoxical preference for hues near the melanopsin activation range. The consistent preference for similar spectral filters observed in AMD and LHON patient groups may reflect a deeper, disease-independent adaptation to mitochondrial stress within the visual system.

Recent neuroimaging studies have shown that patients with LHON exhibit significant cortical reorganization in response to central vision loss, including altered activation patterns in the visual cortex and associated networks^42^. This plasticity may modulate sensitivity to specific visual stimuli or alter the perception of discomfort, potentially aligning with the spectral characteristics of the selected filters. In this context, the filters may provide not only optical relief but also neurofunctional optimization, matching the reorganized sensory processing pathways. Recent neuroimaging studies have demonstrated that patients with AMD, like those with LHON, exhibit signs of cortical reorganization in response to central vision loss^43^. Functional MRI data reveal that the primary visual cortex (V1), which typically processes central visual input, can be recruited to process peripheral stimuli in AMD patients. This suggests that the brain engages neuroplastic mechanisms to adapt to the loss of foveal input, potentially enhancing the use of peripheral vision for visual tasks. Such cortical remapping supports the hypothesis that comfort-tint filters may interact with these reorganized visual pathways, aligning with altered sensory processing to improve visual comfort and function.

Although this study did not include structural or genetic data, the observed clustering of preferred hues raises the possibility of underlying correlations with clinical parameters. For instance, it is plausible that color preferences may relate to the extent of retinal ganglion cell loss, as measured by OCT (e.g., GCC thickness)^44^, or to specific mitochondrial DNA mutations in LHON. Similarly, visual field characteristics from perimetry could influence the subjective experience of comfort or glare. While such correlations could not be explored in the present study due to data limitations, they represent a promising direction for future research. Integrating functional, structural, and genetic data may help elucidate the mechanisms behind individual filter preferences and optimize personalized interventions.

To our knowledge, no questionnaire exists to evaluate specifically visual comfort. Both the LHON and AMD group suffer from photophobia, an inherent symptom of both eye diseases. Photophobia is an apparent reason for reduced comfort during everyday activities. Comfort-tint reduced photophobia in both groups according to the photophobia questionnaire, most in the LHON group, who displayed more photophobia symptoms compared to AMD. The LHON patients also cited improved comfort more frequently than the AMD patients, maybe due to less photophobia with comfort filters. The VLSQ-8 questionnaire was administered before and after one year of comfort-tint use. Quantitative analysis revealed a statistically significant reduction in symptoms among LHON patients, with scores decreasing from a mean of 23.2 at baseline to 19.0 at follow-up. This suggests a clinically meaningful improvement in light sensitivity. In contrast, AMD patients showed a non-significant reduction, with scores changing from 15.0 to 12.8, indicating a more modest effect.

Quantitative results based on the interview showed statistically significant differences in perceived benefits between AMD and LHON, which were supported by qualitative narratives. While AMD participants reported more frequent and task-specific use, emphasizing visual clarity, LHON participants emphasized comfort and psychosocial relief. This divergence may reflect not only the distinct pathophysiology of the two conditions but also differing expectations and coping strategies, offering important insights into the subjective dimensions of visual rehabilitation.

The fact that AMD participants were more likely to report improved vision, yet still preferred filters for comfort, suggests that visual clarity alone does not fully capture the value of the treatment. It may be that for AMD patients, comfort-tints reduce visual stress or fatigue during specific tasks, even if they do not objectively enhance acuity. This aligns with the broader concept of “functional vision”, how vision is experienced in real-world contexts, which is often underrepresented in clinical metrics. In contrast, LHON participants, who typically experience sudden and profound central vision loss, may rely more heavily on non-visual cues and compensatory mechanisms. Their preference for continuous use and emphasis on comfort and concealment may reflect a need for sensory stability and social adaptation. The filters may serve not only to reduce photophobia but also to provide a sense of control or normalization in daily life. This psychosocial dimension, the feeling of being less “visibly impaired”, was repeatedly mentioned in interviews and deserves further exploration. The willingness of participants to continue using the spectacles and even pay for them underscores their perceived benefit. Moreover, the consistency of color preferences over time, as shown by the repeatability of comfort-tint selections, suggests that these choices are stable and meaningful rather than arbitrary. This supports the clinical utility of individualized colorimetry in identifying optimal filters for each patient.

The between session chromaticity differences ($\Delta u' \ v'$) for Comfort 1 and Comfort 2 were small and within the range of repeatability reported by Aldrich^45^ (0.043), despite the substantially longer interval in our study. All values remained below  0.07, a level previously identified as the point at which chromatic deviations reduce the functional effect of a tint. This indicates that comfort tint selections exhibit good stability over time.

Finally, the distinct patterns of use between groups highlight the need for personalized rehabilitation strategies, we suggest that individualized precision-tinted spectacles may offer a valuable adjunct to conventional low vision rehabilitation. A one-size-fits-all approach to filter prescription may overlook critical individual differences in visual needs, lifestyle, and adaptation. The data here suggest that comfort, confidence, and social ease should be considered in therapeutic outcomes. Importantly, visual comfort should be prioritized alongside traditional visual functions such as acuity and contrast sensitivity, as it directly impacts functional vision. Future studies should explore how factors such as disease duration, psychological resilience, and environmental context shape the perceived value of comfort-tints.

This study suffers from several limitations. Although an a priori power analysis was performed, the actual sample size was smaller than required to achieve the desired statistical power. This limitation reflects the exploratory nature of the study, which aimed to provide preliminary insights and inform future research with larger samples. The study groups experienced several dropouts, particularly the AMD group, where four patients failed to return. This makes statistical inference difficult. A notable confounding variable in the comparison between the groups is the difference in age and life stage. Participants in the LHON group were generally younger and actively employed, whereas those in the AMD group were older and predominantly retired. These demographic differences may influence both functional outcomes and psychosocial responses and should be considered when interpreting the results. The follow-up discussion with patients has provided us with many new insights into the concept of vision comfort and visual function. We did not evaluate metrics such as working capacity and Quality of Life, with a focus on visual function. We also did not elaborate on brain fatigue or cognitive aspects of low vision, even though we now believe this could explain some of the positive subjective effects found.

In conclusion, both patient groups preferred precision-tinted glasses within the green-turquoise spectrum for improved comfort, while enhanced vision revealed more bluish hues. This is totally different from traditional yellow edge filters prescribed for low vision. Visual function did not improve with any of the filters but reduced the photophobia according to the VSQL-8 questionnaire, which was especially clear for the LHON group. The study shows the importance of integrating visual comfort considerations into clinical practice.

## Methods

Patients with central visual field reduction due to AMD or LHON were recruited and invited from the low vision clinic in Stockholm to the Marianne Bernadotte Center (MBC) at Karolinska Institutet, located at St. Erik Eye Hospital. Ten patients with AMD (5M/5F, 78.2 years old, S.D. ± 7.5) and eleven patients with LHON (8M/3F, 43.5 years old, S.D. ± 20.7) were invited to participate. Participants were included if they had stable stage AMD or LHON, had documented central visual field reduction, could provide informed consent, and complete colorimetry testing; those with ocular pathology contraindicating spectacle wear or cognitive impairment were excluded. All enrolled participants had a stable form of the disease at baseline, which minimizes variability and potential confounding factors. The total testing time was approximately one hour. The study adhered to the tenets of the Declaration of Helsinki and was approved by the Swedish Ethical Review Authority (Etikprövningsmyndigheten), approval number EPN 2023 06887 01. Written informed consent was obtained from all participants prior to inclusion in the study.

### Study design

All patients were invited to the first visit (Visit 1) and underwent tests before ordering comfort-tint spectacles. An interim telephone assessment was conducted 4–6 weeks after delivery of the comfort-tint spectacles. The purpose was to monitor initial adaptation, identify any adverse effects, and address patient questions regarding lens use. Before the follow-up meeting (Visit 2), all participants had worn the comfort filters for at least 10 months. The extended follow-up period of one year was chosen to ensure evaluation under varying environmental conditions, particularly seasonal differences in daylight exposure in Sweden. The Intuitive Colorimeter was tested to optimize vision (vision-tint) and then retested for comfort (comfort-tint 2). See Table 3 for an overview of assessments.

**Table 3:** Summary of clinical and functional assessments across study visits.

Assessment	Visit 1	Visit 2
Clinical evaluation (slit-lamp, refraction, etc.)	y	y
VLSQ-8 questionnaire	y	y
Intuitive Colorimeter Comfort-tint	y	y
Intuitive Colorimeter Vision-tint	n	y
Visual function test (acuity, contrast) with, without tints and glare	y	y*
Interview	n	y

Tests were conducted on each of the two visits. * = if vision-tint yielded a different tint than comfort-tint.

### Clinical examination

A slit-lamp examination was conducted to evaluate the anterior segment of the eye, ensuring no contraindications for further clinical evaluation. Habitual spectacle correction for distance from the low vision clinic was used to test binocular corrected distance visual acuity, if made recently. If habitual spectacles were not made recently, a subjective over-refraction was performed to assess refraction. If optimal refraction differed from habitual, or if habitual correction had filters, a new correction was placed in a trial frame and used during testing. If patients did not have recently made habitual spectacles, a full subjective refraction was performed to determine optimal correction.

### Subjective symptoms

VLSQ-8 has been utilized to assess the presence and severity of photosensitivity symptoms. After a written agreement with the rights holder, the authors translated the questionnaire into Swedish. The translated version was back-translated into English by a native English speaker to optimize language agreement between the versions. Cronbach’s alpha for the VLSQ-8 responses was above 0.8, indicating a high reliability of the questionnaire. Fewer symptoms result in lower scores on the questionnaire. The VLSQ-8 was administered twice, at visits 1 and 2.

### Intuitive colorimeter

**Fig. 2:**
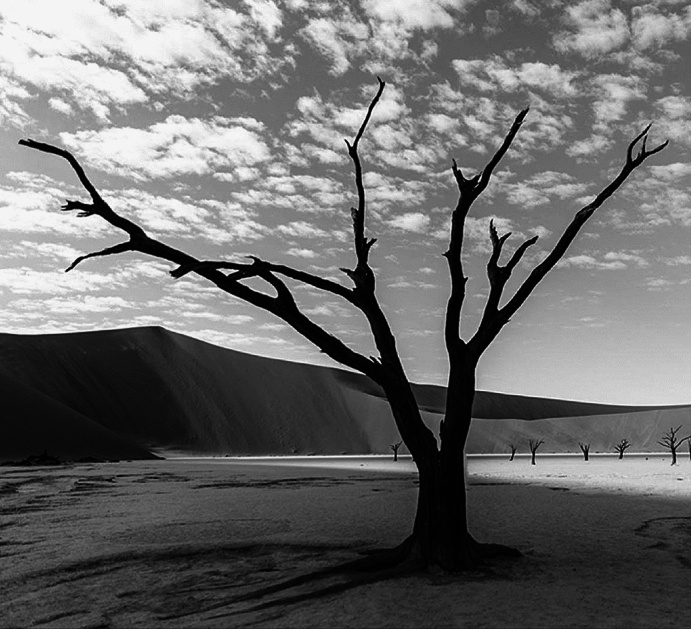
“Dead Vlei Namibia” by Josef Bürgi (used with the photographer’s permission).

The apparatus was placed on an adjustable table. The image “Dead Vlei Namibia” by Josef Bürgi (Fig. 2) was used with the photographer’s permission as a visual fixation target within the apparatus (approximately 45 cm fixation distance). This image allows attending to a visible object despite central visual field defects and enhances focus during colorimetry testing.

Participants were instructed to look at the tree and observe the colored illumination within the surrounding space. The testing principle involved comparing colored light from the 12 color segments (saturation 30) on the 360-degree chromaticity wheel to white light. Each color was evaluated as either good, indistinguishable from, or worse than white light. Following this initial round, a shortlist of favourable colors remained. Color saturation was tested for each color; if no difference was detected, saturation was set to 30. Each color was then finely tuned by adjusting the hue. The subject was asked to identify any differences between a specific shift in either a positive or negative direction around the current hue. A preference in any direction prompted an adjustment in color preference accordingly. This process was repeated until no difference was observed between the two conditions. In the final step, each color was tested under full and half illumination. The accompanying software then calculated the final precision tint hue based on the adjusted shortlist.

### Precision tint optimized for comfort or vision

Testing was done for two conditions: to optimize comfort (comfort-tint) or vision (vision-tint). The only difference between them was the instructions given to the patient during colorimetry testing. For comfort-tint, the instruction was to indicate which condition was most comfortable, regardless of any improvement in vision. Clear instructions were needed and repeated to emphasize the focus on comfort. For vision-tint, the instruction was to indicate which hue yielded the best visual appearance, focusing on sharpness and clarity, regardless of comfort or discomfort. After colorimetry, the filter combination was mounted in a trial frame from the lens box of colored trial lenses and tested on the patient. The spontaneous subjective response was noted while viewing the examination room and a room with normal daylight. After confirming whether a positive filter effect was present, a decision was made regarding the ordering of comfort-tint spectacles. The lenses were ordered, after choosing a frame, with correction for distance vision (if needed) and the comfort-tint from Cerium Vision Technology in the United Kingdom. The lenses were sent to Sweden to be cut and mounted in the frames before being delivered to MBC. Participants received the glasses free of charge. Patients were not provided with any requirements concerning the frequency and conditions for wearing glasses; instead, they received verbal recommendations for usage based on their needs.

### Visual function

The procedure for determining visual acuity was conducted following a standardized testing protocol using ETDRS charts (100% contrast at 3m and 10% contrast at 2m) in an illumination cabinet (Precision Vision). The cabinet was moved from the intended position toward the patient to counter the low visual function, ensuring that the first line was readable, after which all testing for that chart was carried out at that distance. Patients were instructed to identify as many letters as possible, even if that involved guessing. When no letter was correctly identified on one line, testing was aborted. The exact eye-chart distance was controlled and noted using a ruler during the examination. LogMAR acuity was then recalculated for each patient according to the reading distance between the eye and the acuity chart in meters. Testing of glare was conducted only on 100% contrast charts using a glare source with halogen lamps attached to the illumination cabinet (Precision Vision). All testing were repeated using no filters or preferred colored filters.

### Follow-up meeting (visit 2)

After approximately one year of wear, all participants were again invited for follow-up testing and interviews. The follow-up meeting began with the Intuitive Colorimeter to optimize visual function (vision-tint). This was done using neutral spectacle lenses (no filters) in a trial frame if needed. If the Colorimeter resulted in a different tint than the comfort-tint 1, a separate visual function test using the vision-tints was included in this meeting. The VLSQ-8 questionnaire was administered, followed by a comprehensive interview including closed and open-ended questions regarding the use of the comfort-tint spectacles. At the end of this meeting, the Intuitive Colorimeter was again administered to test the repeatability of the comfort measurement (comfort-tint 2). An in-depth interview was conducted using a structured protocol to evaluate the use and effectiveness of the precision-tint spectacles. This protocol included closed and open-ended questions to collect comprehensive participant feedback (see Table 4).

**Table 4:** Interview questions about the use of comfort-filter spectacles.

Interview questions	Response format
1. How often do you use the glasses?	Days per week / Hours per day
2. Do you experience better vision?	Yes / No
3. Do you experience better comfort?	Yes / No
4. Are the glasses sufficient in intense light?	Yes / No
5. When do you use them?	Open-ended
6. Why do you use them?	Open-ended
7. What happens when you take them off?	Open-ended
8. What is the difference compared to your previous filters/sunglasses?	Open-ended
9. Will you continue to use the glasses after the study is completed?	Yes / No
10. Would you do it if you had to pay for these glasses yourself?	Yes / No
11. Would you choose glasses with filters for better vision or better comfort?	Vision / Comfort

The interview included both closed-format and open-ended questions to explore participants’ experiences.

### Data and statistics

An a priori power analysis was conducted using G*Power 3.1^46^ for a point biserial correlation (t-test family) with the following parameters: effect size $|\rho | = 0.30$ (medium effect according to Cohen), $\alpha = 0.05$, power $(1-\beta ) = 0.95$, and a one-tailed test. The calculation indicated that a total sample size of $N = 111$ participants would be required to achieve the desired power. This study included 23 participants, which corresponds to an achieved power of approximately 43%. Comfort-tint and vision-tint data were evaluated through angular hue values in degrees on the chromaticity wheel. The analysis of differences between conditions and groups required circular statistics to ensure appropriate comparisons (since ${0}^\circ$ and ${360}^\circ$ represent the same hue). The non-parametric Mardia-Watson-Wheeler test was used to statistically compare conditions within and between groups. R statistics (Packages: Circular, CircStats) were used for analysis^47^.

Recalculated visual acuity data were analyzed using a repeated measures ANOVA model after confirming the appropriateness of parametric statistics. Interview responses, including numerical data, were analyzed and presented with descriptive statistics. The Mann-Whitney U test was used for further analysis of quantitative responses. Qualitative answers were summarized for each patient group.

## Data Availability

The datasets generated and analyzed during the current study are available from the corresponding author on reasonable request.
